# The distribution of myeloid-derived suppressor cells subsets and up-regulation of programmed death-1/PD-L1 axis in peripheral blood of adult CAP patients

**DOI:** 10.1371/journal.pone.0291455

**Published:** 2023-09-27

**Authors:** Haihong Gong, Jingquan Zhao, Wenshuai Xu, Yinghua Wan, Xiangdong Mu, Mingqiang Zhang

**Affiliations:** 1 Department of Respiratory and Critical Care Medicine, Affiliated Hospital of Qingdao University Medical College, Qingdao, China; 2 Department of Respiratory and Critical Care Medicine, Tsinghua Changgung Hospital, School of Clinical Medicine, Tsinghua University, Beijing, China; University of Gondar, ETHIOPIA

## Abstract

**Background:**

Myeloid-derived suppressor cells (MDSCs) have been reported to expand and have a potent ability in the expansion of regulatory T cells in malignant and infectious disease. The current study was performed to investigate the role of MDSCs and possible immune mechanisms in dampening immune responses of community acquired pneumonia (CAP).

**Methods:**

This was a single-center cross-sectional study. The distribution of MDSCs subsets, the PD-1/PD-L1(L2) level of MDSCs subsets and Tregs in the peripheral blood of adult CAP patients and healthy control were measured by flow cytometry analysis.

**Results:**

Peripheral blood mononuclear cells (PBMCs) from 63 adult CAP patients contained an elevated frequency of both G-MDSC (4.92±0.30 vs 2.25±0.21,p<0.0001) and M-MDSC (19.40±1.30 vs 9.64±0.57,p<0.001) compared to healthy controls. Treg in the peripheral blood of CAP patients exhibited increased expression of PD-1 and CTLA-4, accompanied by no difference of their frequency. Moreover, up-regulated expression of PD-L1 on MDSC subsets in the peripheral blood of CAP patients was also revealed. Of note, the frequency of circulating MDSCs subset displayed a positive correlation with neutrophil count percentage in blood in CAP patients.

**Conclusions:**

In summary, the significant expansion of circulating MDSCs subsets and the up-regulated expression of PD-1/PD-L1 level in CAP patients may suggest the possible involvement of PD-1/PD-L1axis in MDSCs mediated immune regulation on Treg at least partially in CAP patients.

## Background

Community-acquired pneumonia (CAP) is still an important infectious cause of mortality and morbidity worldwide caused by a wide variety of micro-organisms including bacteria, fungi and virus [1]. The host innate and adaptive immune responses play crucial roles in the pathological processes of CAP and determine disease severity [2–4]. Previous studies have demonstrated that the dysregulation of T cells, neutrophils and macrophages are involved in CAP [2–4]. Recent research indicates that myeloid-derived suppressor cells (MDSCs) also play key roles in maintaining pulmonary homeostasis [5, 6] and the role of MDSCs in CAP has not been fully elucidated.

MDSCs are a heterogeneous population of cells that include myeloid progenitor cells, immature granulocytes and macrophages [7, 8], which have gained great attention recently. Under pathological conditions such as tumor environments, MDSCs are often strongly expanded [7, 8]. Human MDSCs are divided into two subsets based on their surface markers’ expression: monocytic MDSCs (M-MDSCs) and granulocytic MDSCs (G-MDSCs) [7]. First described in malignant diseases, the role of MDSCs in infectious disease conditions has not been well studied [9, 10]. Several bacteria(both Gram-positive and -negative bacteria) and viruses have been shown to modulate MDSCs [11, 12]. MDSCs have strong immunosuppressive capability of suppressing T cells responses. MDSCs have been shown to promote the expansion of regulatory T cells(Treg), which also exhibit cross-talk with MDSCs [7, 9]. Some researchers have revealed that Tregs regulate the immunosuppressive function of MDSCs [13].

MDSCs can regulate the function of Treg through several mechanisms, including expression of arginase-1, inducible nitric oxide synthase and up-regulation of PD-1/ PD-L1 [9]. Programmed death-ligand 1 (PD-L1) is expressed extensively on immune cells (e.g MDSC) and could suppresses the activation of T-cells upon binding to its receptor PD-1. Recently, several studies have suggested that the expression of PD-L1 on MDSCs is indicative of their immunosuppressive function [14]. Previous study revealed that the proportion of PD-L1^+^ MDSCs was significantly higher in malignant diseases [15]. Subsequent studies also highlighted the high expression level of PD-L1 on MDSCs in infectious diseases [16].

However, few studies have demonstrated the distribution of the subsets of MDSCs and the expression of PD-1/PD-L1 in CAP patients. The current study aims to figure out the distribution of circulating MDSCs subsets and whether PD-L1/PD-1axis is involved in CAP patients.

## Methods

### Study population

This was a single-center cross-sectional study. 63 adult CAP patients from Tsinghua Changgeng Hospital and 24 age-matched healthy donors considered controls were enrolled between February 1, 2019 to August 1,2019 in this study. The diagnosis of CAP was based on clinical presentation and chest radiograph. Patients with asthma, chronic obstructive pulmonary disease, pulmonary tuberculosis, lung cancer and interstitial lung disease were excluded. The clinical parameters in the blood which consisted of erythrocyte sedimentation rate (ESR), and C-reactive protein (CRP), leukocyte count, neutrophil count percentage, lymphocyte count percentage were obtained from patients of CAP. Patient characteristics are shown in Table 1. The studies were reviewed and approved by the Ethics Committee of the Tsinghua Changgeng Hospital(No.18190-0-01).We collected the patient information in strict confidence and used the remaining samples of the patients after testing. So we applied for a waiver of informed consent which was approved by the Ethics Committee of the Tsinghua Changgeng Hospital.

**Table 1:** Characteristics of all subjects.

Variables	healthy controls	CAP
Subjects (No.)	24	63
Age (year)	64.0±7.3	67.6±14.6
Gender (male/female)	8/16	18/45
Leukocytes (×10^9^/liter)	-	9.7±3.69
Percentage of neutrophils in leukocytes (%)	-	75.5±10.67
CRP (mg/L)	-	56.69±44.62

Notes: The data are presented as mean ± standard error of mean (SEM) or mean (range). Abbreviations: CAP, community-acquired pneumonia

### Cell collection

Heparinized peripheral blood samples were obtained from each subject and PBMCs were isolated by density-gradient centrifugation as previously described.

### Flow cytometry

Freshly obtained human PBMC were stained with the following antibodies for 30mins at 4°C: (FITC, PE, PerCP-Cyanine5.5, APC)-conjugated Abs (anti-CD3, CD4, CD8, CD15,CD33, CD11b,CD14, CD127,HLA-DR,CD15,PD-1, PD-L1,CTLA-4).The gated strategies to identify T cells subpopulations and MDSCs subsets, and M-MDSCs (CD14^+^ CD15^-^ CD11b^+^ CD33^+^HLA-DR^-/low^) and G-MDSCs (CD15^+^CD33^+^ CD11b^+^ CD14^-^ HLA-DR^-/low^) were shown below respectively. Tregs were identified by gating on the CD3^+^CD4^+^ CD25^+^ CD127^-/low^ cell population. All antibodies were purchased from eBioscience (Invitrogen, USA)and the corresponding isotype were used in all experiments. After washing the cells with PBS, the data of Flow cytometry were acquired using FACSCalibur^TM^ flow cytometer and analyzed with FlowJo software immediately. The Flow cytometry results were all shown as percentages of gated cells.

### Statistical analysis

All data analyses were performed by GraphPad PRISM software version 8. Normally distributed data were analyzed by Student’s t test(two groups) to assess differences. Correlation was evaluated with Spearman rank correlation. p value less than 0.05 was considered as statistically significant. * for p< 0.05,** for p<0.01, *** for p<0.001, **** for p<0.0001.

## Result

### Demographic characteristics of study population

Clinical characteristics of CAP patients and controls are presented in Table 1.

### CAP patients exhibit elevated levels of circulating MDSC subsets

**Fig 1:**
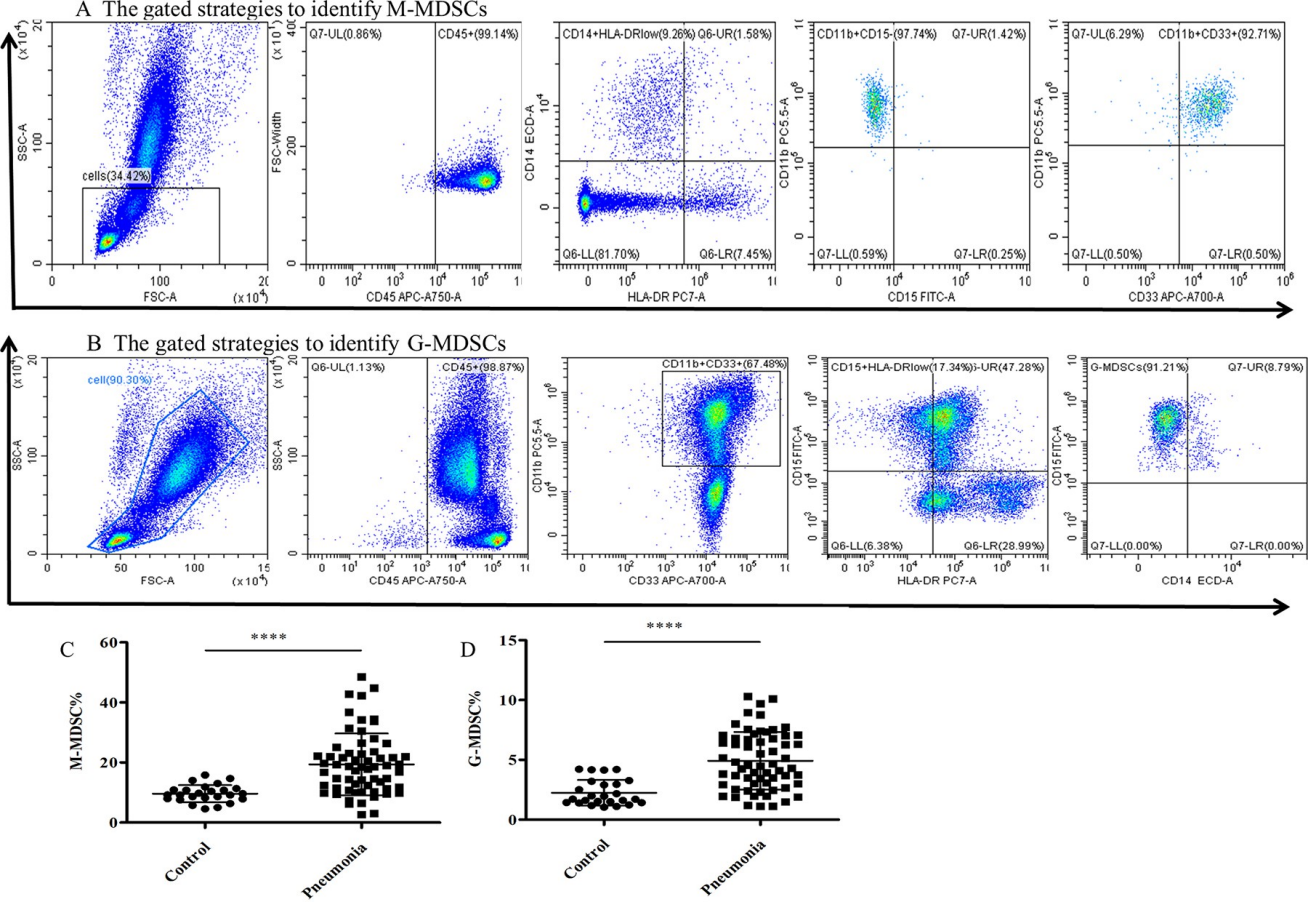
The frequency of MDSCs subsets in peripheral blood of CAP patients. (A)The gated strategies to identify M-MDSCs.(B)The gated strategies to identify G-MDSCs.(C)The percentage of M-MDSCs in the peripheral blood from 24 healthy controls and 63 CAP patients.(D) The percentage of G-MDSCs in the peripheral blood from 24 healthy controls and 63 CAP patients. ****p<0.0001.

The frequency of circulating MDSC subsets was measured by flow cytometry analysis. Using our previously staining strategy to discriminate MDSC subsets, G-MDSC and M-MDSC(Fig 1A and 1B). We found that PBMCs from CAP patients contained an elevated frequency of both and M-MDSC(19.40±1.30 vs 9.64±0.57,p<0.001) and G-MDSC (4.92±0.30 vs 2.25±0.21,p<0.0001) compared to healthy controls (Fig 1C and 1D).

### Treg in the peripheral blood of CAP patients exhibited increased expression of PD-1 and CTLA-4, accompanied by no difference of their frequency

**Fig 2:**
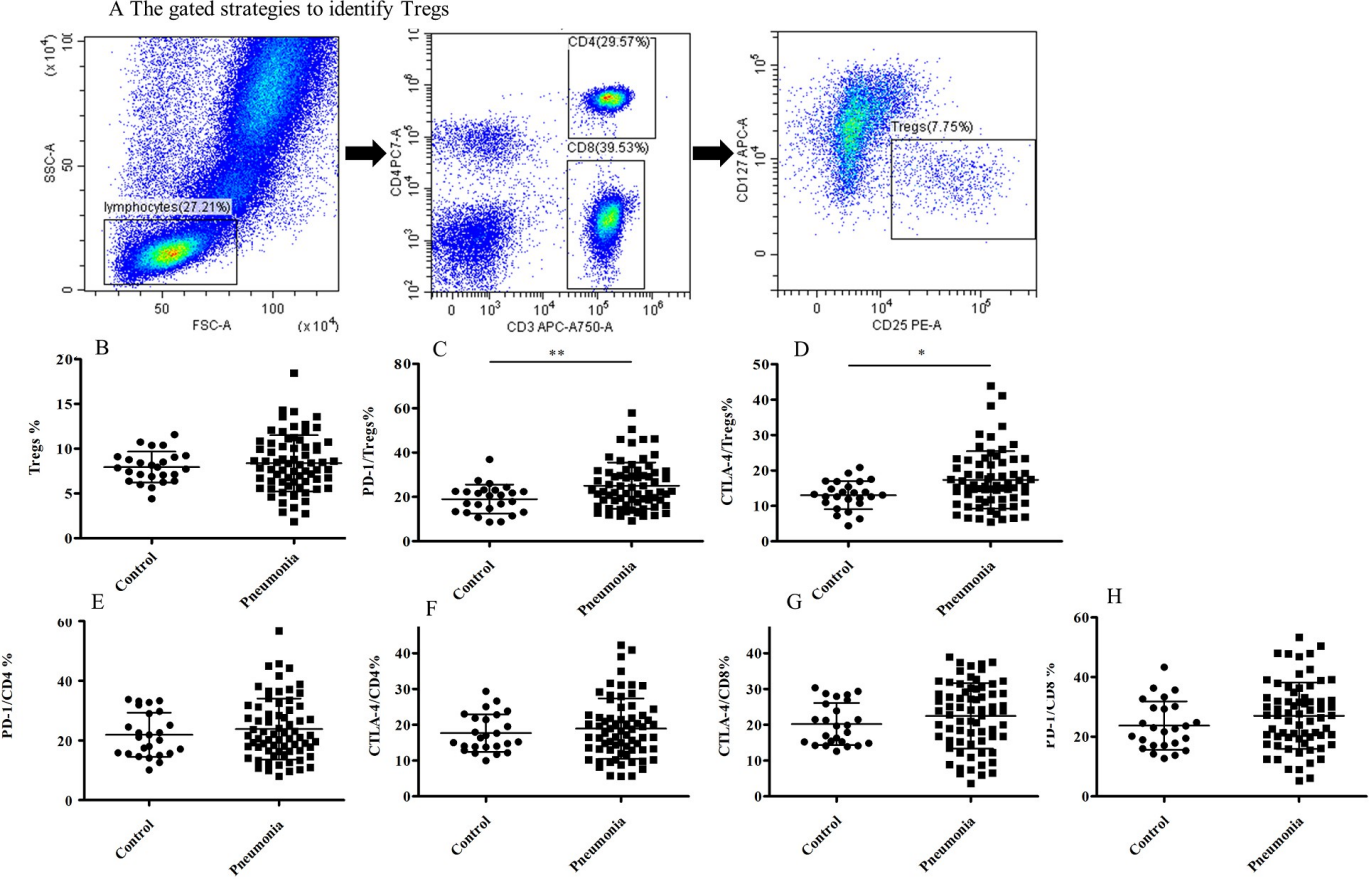
Increased expression of PD-1 and CTLA-4 on Treg in CAP, without no significant difference in the frequency of Treg. (A)The gated strategies to identify Tregs. (B)FACS analysis of Treg cells in peripheral blood from 24 healthy controls and 63 adult CAP patients. (C-D)FACS analysis of surface expression of PD-1 and CTLA-4 on Treg in peripheral blood from24 healthy controls and 63 adult CAP patients. (E-F)FACS analysis of surface expression of PD-1 and CTLA-4 on CD4^+^Tcells in peripheral blood from24 healthy controls and 63 adult CAP patients.(J-H)FACS analysis of surface expression of PD-1 and CTLA-4 on CD8^+^Tcells in peripheral blood from24 healthy controls and 63 adult CAP patients. *p<0.05.**p<0.01.

MDSCs are critical in regulating immune responses by suppressing T cells, including Treg. However, no significant difference in the proportion of the Treg cells was found between CAP patients and healthy individuals (Fig 2A and 2B). To determine whether Tregs from CAP patients exhibited the potential for high immunosuppressive capacity, we investigated the expression of exhaustion marker PD-1 and CTLA-4 on Treg next. Our result shows that, in CAP patients, the percentages of PD-1^+^ and CTLA-4^+^ Tregs were significantly higher than those in healthy control (p<0.001 and p<0.05, respectively (Fig 2C and 2D).

However, there was no significant difference in the expression of PD-1 and CTLA-4 on both CD4^+^ T cells and CD8^+^ T cells between healthy individuals and CAP patients (Fig 2E–2H).

### Altered expression of PD-L1(CD274)and L2(CD273) expression on MDSC subsets in peripheral blood of CAP patients

**Fig 3:**
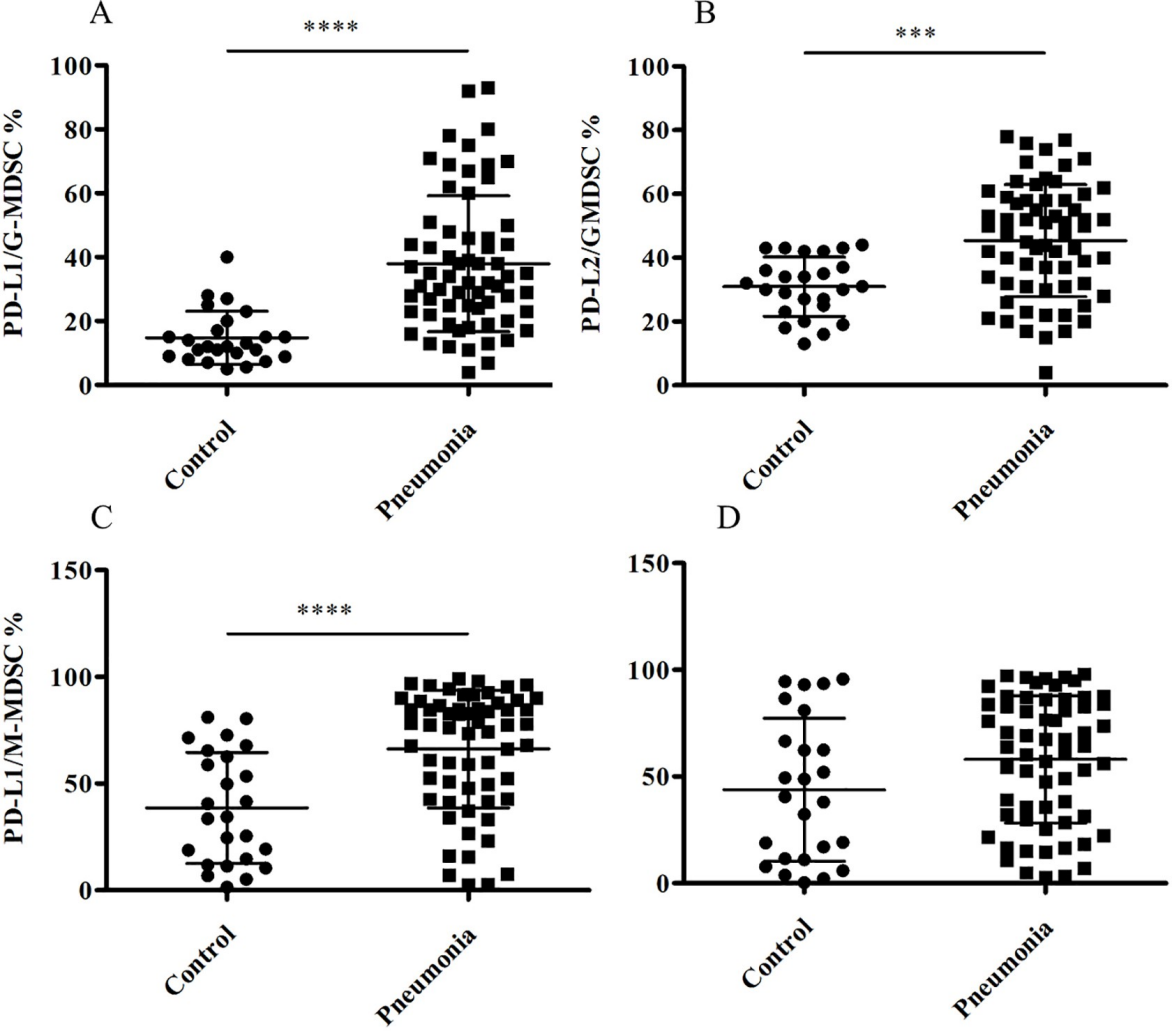
Altered expression of PD-L1 and PD-L2 expression on MDSC subsets in peripheral blood of CAP patients. (A)FACS analysis of surface expression of CD274 (PD-L1) on G-MDSCs in peripheral blood from 24 healthy controls and 63 adult CAP patients.(B) FACS analysis of surface expression of CD273 (PD-L2) on G-MDSCs in peripheral blood from 24 healthy controls and 63 adult CAP patients. (C)FACS analysis of surface expression of CD274 (PD-L1) on M-MDSCs in peripheral blood from 24 healthy controls and 63 adult CAP patients. (D)FACS analysis of surface expression of CD273 on M-MDSCs in peripheral blood fro m 24 healthy controls and 63 adult CAP patients.***p<0.001, ****p<0.0001.

PD-L1 and PD-L2 are the natural ligands of PD-1,which negatively regulates the suppressive function of T-cells. Considering the increased level of PD-1 on Tregs, we further compared the frequency of PD-L1(L2)^+^ MDSC subsets between the two groups. We also found that G-MDSC expressed higher percentage of PD-L1 and PD-L2 in comparison to the control group(p<0.001 and p<0.0001, respectively, Fig 3A and 3B). Moreover, PD-L1^+^ M-MDSC were markedly higher in CAP patients than in the matched peripheral blood of healthy individuals(p<0.0001, Fig 3C). In contrast, there was no significant difference in the frequency of PD-L2^+^ M-MDSCs between healthy individuals and CAP patients(Fig 3D).

### MDSCs frequency in blood is correlated with clinical parameters in CAP patients

**Fig 4:**
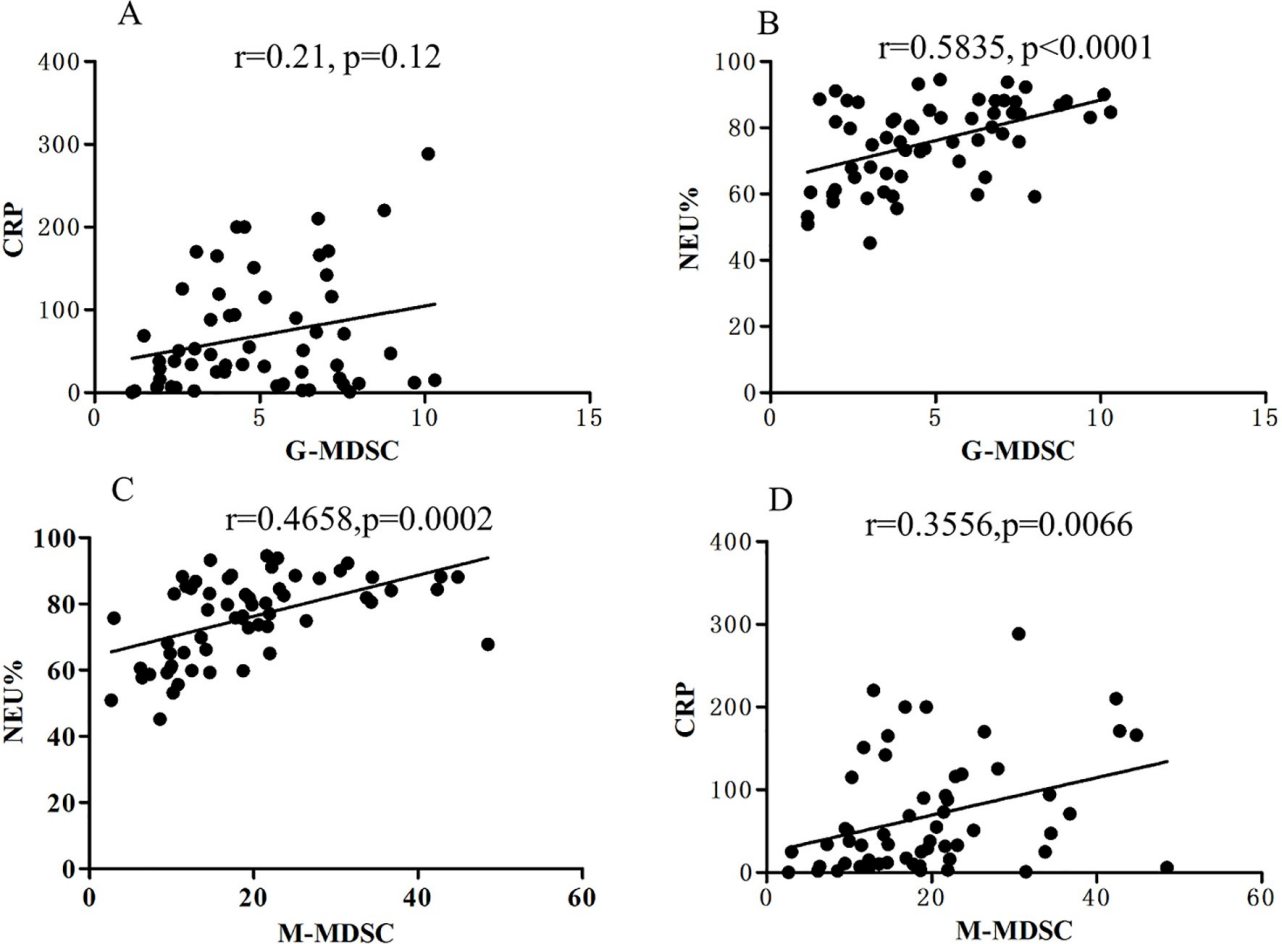
MDSCs frequency in blood is correlated with clinical parameters in adult CAP patients. (A)correlations between the frequencies of G-MDSCs and c-reactive protein (CRP) level in blood of 63 CAP patients.(B) correlations between the frequencies of G-MDSCs and neutrophil percentage in peripheral blood from 63 CAP patients. (C) correlations between the frequencies of M-MDSCs and neutrophil percentage in peripheral blood from 63 CAP patients. (D) correlations of the frequencies of M-MDSCs and CRP level in blood of 63 CAP patients.

To gain further insight into the role of MDSCs in CAP pathogenesis, we analyzed the potential association between the frequency of MDSCs subsets in blood and clinic parameters. Our results revealed that neutrophil percentage and C-reactive protein (CRP) level and positively correlated with circulating M-MDSC frequency (r = 0.5835, p<0.001, and r = 0.3556, p<0.01, respectively, Fig 4A and 4B. Moreover, neutrophil count frequency positively correlated with G-MDSC percentage in peripheral blood (r = 0.4658,p < 0.001, Fig 4C). However, we did not find a significant correlation between G-MDSC percentage and CRP level in CAP patients (Fig 4D).

## Discussion

Myeloid-derived suppressor cells (MDSC) are an immuno-suppressive subpopulation of myeloid cells that have potent ability to suppress T cell activity and induction of regulatory T cells (Treg) in many pathological conditions as well as in some physiological settings [17]. However, the role of MDSCs and the cross-talk between MDSCs and Treg have not been fully elucidated in CAP. In this present study, we found that CAP patients exhibit an elevated frequency of circulating MDSC subsets, accompanied by a significant upregulation of PD-1 and CTLA-4 on Treg, but no difference in Treg frequency. We indicate that MDSC may regulate Treg function in CAP patients. Moreover, increased level of PD-L1(L2) on MDSC subsets were found in CAP patients and thus, speculating, the function of MDSCs may be activated. Collectively, these findings may suggest that the PD-1/PD-L1 axis may be involved in MDSCs-mediated immune regulation on Treg at least partially in CAP patients.

The role of MDSCs subsets in CAP were relatively inadequate than malignant conditions and inconsistent up to date. Human MDSC were identified as two major types: granulocytic (G-MDSC) and monocytic (M-MDSC).An increased frequency of both M-MDSC and G-MDSC was detected in patients of CAP compared to healthy donors in the current study, which is in line with previous studies in patients with autoimmune disease [18], cancer [19, 20] and many infectious diseases [19, 21]. MDSCs considerably suppress T cell activation and function, including Treg [11]. Oddly enough, we did not detect a significant difference in the proportion of the Tregs between CAP patients and healthy individuals. The lack of difference in Tregs frequency may be explained as follows: first, the proportion of the Tregs may be associated with the stage and the severity of pneumonia [22–24]; second, PD-1 and CTLA-4 are important negative immune regulators expressed on regulatory T cells and play an important role in maintaining the function of Treg cells [25]. The increased expression of PD-1 and CTLA-4 on Treg in our study was expected considering the immunomodulatory effects of MDSC on Treg. Therefore, it is possible that MDSC may mainly regulate Treg function in CAP patients.

PD-L1 and PD-L2 are the two ligands of PD-1which are also expressed on MDSC. Previous studies demonstrated that percentages of MDSCs expressing PD-L1 and PD-L2 were significantly increased and MDSC may regulate T cells by PD-1/PD-L1 [16, 26, 27]. Lei et al revealed that myeloid-derived suppressor cells impair alveolar macrophages through PD-1 receptor ligation during Pneumocystis pneumonia [16, 28]. To the best of our knowledge, the PD-L1(L2) expression on MDSCs is relatively rare in previous studies in CAP patients. Our result revealed that markedly increased expression of PD-L1 on both M-MDSC and G-MDSC in CAP patients than in the matched peripheral blood of healthy individuals. Therefore, MDSCs may interact with Treg through PD-1/PD-L1 ligation in CAP patients. However, our conjecture remains to be further confirmed by vitro experiments. PD-L2 is upregulated especially on G-MDSC, the significance of which is still unknown.

Both animal models and all clinical studies to date uncovered that high proportions of MDSCs were associated with clinical worsening in infectious diseases along with sepsis [29, 30]. In line with the previous study, we found that neutrophils percentage is positively correlated with circulating MDSCs subsets frequency [31, 32] and C-reactive protein (CRP) level are especially positively correlated with circulating M-MDSC frequency [33].

The present study also has some limitations. First, our result lacks in vitro experiments and validation to better understand the effects of MDSCs. Second, our sample size was relatively small and generalization of the results should be cautiously considered. Third, the conclusions should be interpreted with caution due to lack of subgroup data.

## Conclusions

Cumulatively, by extensive analyses of 63 patients immunological and clinical parameters along with 24 healthy donors, we observed that CAP patients displayed strong expansion of the MDSC subset, altered expression of PD-1 on Treg and PD-L1 expression on MDSC. However, no significant difference in the proportion of the Treg cells was found between CAP and the matched group. We speculate that the PD-1/PD-L1 axis may be involved in MDSCs-mediated Treg suppressive properties at least partially in CAP patients. Additional investigations are necessary to further endorse the current outcomes.

## List of abbreviations

CAP: community-acquired pneumonia
MDSCs: Myeloid-derived suppressor cells
M-MDSCs: monocytic MDSCs
G-MDSCs: granulocytic MDSCs
Treg: regulatory T cells
PD-L1: Programmed death-ligand 1
ESR: erythrocyte sedimentation rate
CRP: c-reactive protein
PBMCs: peripheral blood mononuclear cells
